# Comparison between Protocols for Management of Fetal Growth Restriction

**DOI:** 10.1055/s-0043-1764493

**Published:** 2023-03-28

**Authors:** Caio Ribeiro Vieira Leal, Karen Pereira Rezende, Evilane do Carmo Patrício de Macedo, Guilherme de Castro Rezende, Mário Dias Corrêa Júnior

**Affiliations:** 1 Universidade Federal de Minas Gerais, Belo Horizonte, MG, Brazil; 2 Diagnostic Imaging Solutions, Belo Horizonte, MG, Brazil

**Keywords:** Fetal growth restriction, Doppler velocimetry, Prematurity, Cardiotocography, Protocols, Restrição de crescimento fetal, Dopplervelocimetria, Prematuridade, Cardiotocografia, Protocolos

## Abstract

This comprehensive review compares clinical protocols of important entities regarding the management of fetal growth restriction (FGR), published since 2015. Five protocols were chosen for data extraction. There were no relevant differences regarding the diagnosis and classification of FGR between the protocols. In general, all protocols suggest that the assessment of fetal vitality must be performed in a multimodally, associating biophysical parameters (such as cardiotocography and fetal biophysical profile) with the Doppler velocimetry parameters of the umbilical artery, middle cerebral artery, and ductus venosus. All protocols reinforce that the more severe the fetal condition, the more frequent this assessment should be made. The timely gestational age and mode of delivery to terminate the pregnancy in these cases can vary much between the protocols. Therefore, this paper presents, in a didactic way, the particularities of different protocols for monitoring FGR, in order to help obstetricians to better manage the cases.

## Resumo

Esta revisão compreensiva compara protocolos clínicos de entidades importantes em relação ao manejo da restrição de crescimento fetal (RCF), publicados desde 2015. Cinco protocolos foram escolhidos para a extração de dados. Não houve diferenças relevantes quanto ao diagnóstico e classificação da RCF entre os protocolos. Em geral, todos os protocolos sugerem que a avaliação da vitalidade fetal deve ser realizada de forma multimodal, associando parâmetros biofísicos (como cardiotocografia e perfil biofísico fetal) aos parâmetros dopplervelocimétricos da artéria umbilical, artéria cerebral média e ducto venoso. Todos os protocolos reforçam que quanto mais grave a condição fetal, mais frequente essa avaliação deve ser feita. A idade gestacional oportuna e o modo de parto para interromper a gravidez nesses casos podem variar muito entre os protocolos. Portanto, este trabalho apresenta, de forma didática, as particularidades de diferentes protocolos de acompanhamento de RCF, a fim de auxiliar os obstetras no melhor manejo dos casos.

## Introduction

Fetal growth restriction (FGR) affects about 10% of pregnancies and is a potentially serious condition that can lead to intrauterine death, intrapartum fetal distress, and admissions to the Neonatal Intensive Care Unit (NICU). 1 2 Advances in fetal monitoring techniques, such as Dopplervelocimetry, have made possible a better understanding of placental and fetal blood flows and, consequently, a better understanding of the natural history of this condition. 3 Through a better knowledge of the disease, new clinical follow-up protocols were published, but it was only in 2014 that Figueras and Gratacós 4 managed to systematize all knowledge into growth restriction categories, with specific suggestions for monitoring, and timing and mode of delivery. After this seminal work, several obstetric entities around the world updated their protocols, incorporating, partially or completely, the suggestions made by Figueras and Gratacós. 4 The aim of this review is to compare clinical protocols for monitoring singleton-pregnancy fetuses, with growth restriction, published from 2015 on, highlighting their similarities and differences. The main focus will be on the diagnosis, management, and time of delivery. Other aspects, such as prevention, therapies, and preparation for delivery will not be addressed.

## Methods

**Fig. 1:**
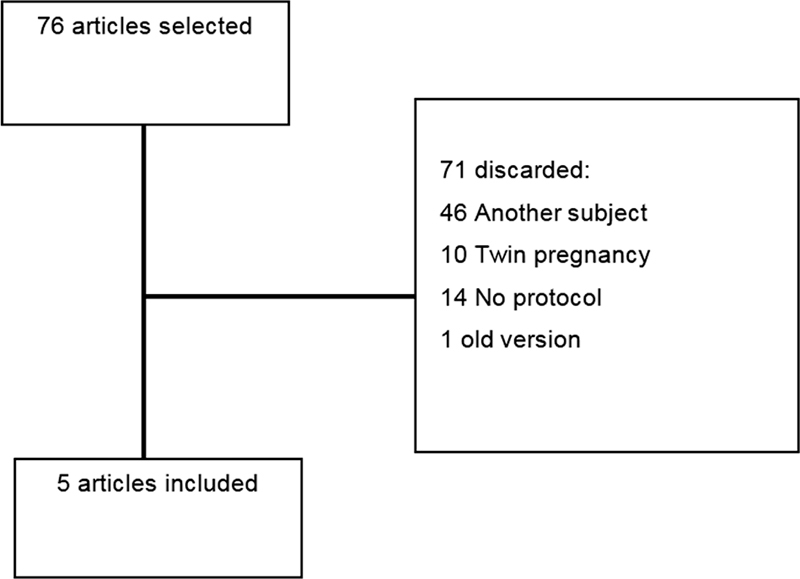
Search and selection of included articles

A search was performed in PUBMED and Scielo with the descriptors *: “Fetal Growth Restriction OR Intrauterine Growth Retardation AND Guideline”* . The search identified 76 articles published between 2015 and 2021. Reading the titles and abstracts enabled the selection of 5 articles that met the proposed criteria ( Figure 1 ). The articles included in the analysis are the International Federation of Gynecology and Obstetrics (FIGO) 2021 protocol, 1 the International Society of Ultrasound in Obstetrics and Gynecology (ISUOG) 2020 protocol, 2 the Society for Maternal-Fetal Medicine (SMFM) 2020 protocol, 3 the French College of Gynecologists and Obstetricians (FCOG) 2015 protocol 5 and the American College of Obstetricians and Gynecologists (ACOG) 2021 protocol. 6

The protocols were reviewed by all authors, who sought to identify similarities and highlight the differences between them, also analyzing the bibliographic references suggested by the authors to support their recommendations.

## Diagnosis

On the other hand, FGR is defined as the failure to reach the fetus' growth potential, caused by a pathological process, most commonly placental insufficiency. 1 2 3 5 6 Diagnosis can be made through physical examination, the fundal height measurement, or ultrasonography, but ultrasonography is the most accurate method for FGR diagnosis, as will be discussed below. 1

## Fundal Height Measurement

The fundal height measurement (FHM) is a simple and inexpensive method that can be widely used as a strategy to track FRG, and should be performed with the patient in the supine position, using an inelastic measuring tape, after emptying the bladder. 1 This measurement must be made following a standardized technique, defined as the distance between the upper border of the pubic symphysis and the fundus of the uterus. 1 The measurement in centimeters, between 24 and 38 weeks, is close to the gestational age. 1 The accuracy of FHM in predicting fetuses whose weight is below the 10th percentile is limited and there are no randomized controlled studies comparing the measurement with serial ultrasounds for the assessment of fetal biometry. 1 A meta-analysis with 34 observational studies showed that FHM has a sensitivity of 58% and specificity of 87% for predicting fetal weight below the 10th percentile. A single measurement between 32 and 34 weeks had between 65 and 85% sensitivity and 96% specificity for detecting growth-restricted fetuses. 1 It is important to emphasize that some factors such as maternal obesity, uterine fibroids, and polyhydramnios may limit FHM as a screening tool for FGR. 1

## Fetal Biometry

Fetal biometry performed through ultrasonography is the cornerstone for detecting fetal growth disorders and includes the assessment of head circumference (HC), biparietal diameter (BPD), abdominal circumference (AC), and femur length (FL). The measurement of these biometric indices must be obtained in a standardized way by an experienced operator. 1 Fetal weight can be estimated using various combinations of these four biometric indices. The accuracy of most equations for calculating estimated fetal weight (EFW) has an estimated error in the range of 10%, and the error is greater at the extremes of fetal weight. EFW can be influenced by factors such as fetal sex, fetal presentation, and number of fetuses. 1 Studies that compared the accuracy of different available equations concluded that those based on a combination of three to four biometric indices provide more reliable and consistent results. A recent systematic review showed that the Hadlock equation, which is based on three indices (HC, AC, and FL) provides the greatest accuracy. 7 Considering that the accuracy of the equations varies among different populations, it would be reasonable to choose those validated within the local population. However, when this is not possible, which is very often, the use of Hadlock's formula should be considered. 1

### Estimated Fetal Growth Assessment Versus Abdominal Circumference Assessment

The most common definition of an SGA fetus is the one whose EFW or AC is below the 10th percentile for its gestational age. This SGA fetus has no increased risk for an adverse perinatal outcome, while the growth-restricted fetus has both increased perinatal and long-term risks. 2 Fetuses whose birth weights are below the 10th percentile are at increased risk of stillbirth and perinatal death and those fetuses with EFW below the 3rd percentile are at even greater risk. For this reason, fetuses with EFW or AC below the 3rd percentile may have one of these parameters used as an isolated criterion for the diagnosis of FGR at any gestational age. 1 2 3 5 6 In a prospective cohort study with 1000 low-risk pregnant women, both AC measurement and EFW below the 10th percentile demonstrated similar diagnostic accuracy to predict SGA fetuses. 3 In a meta-analysis published in 2017, AC and EFW below the 10th percentile predicted SGA fetuses with similar sensitivity and specificity. When AC below the 5th percentile was used as a cutoff point, it was found lower sensitivity, but greater specificity in predicting SGA fetuses. 3 According to the SMFM, FGR is defined as EFW or AC measurement below the 10th percentile for gestational age. 3 According to the ISUOG, one isolated measure of EFW or AC is not sufficient to identify fetuses with growth restriction unless the EFW or AC are below the 3rd percentile. The criteria used by SMFM compared to those described by ISUOG in the diagnosis of FGR have a greater sensitivity in predicting low birth weight infants and their neonatal complications (54.7 versus 28.8%), but a higher false positive rate (6.7 versus 1.6%). 2 It is important to point out that, when the diagnosis of FGR is made, a thorough obstetric examination is recommended, due to the fact that in up to 20% of cases, the restriction is associated with fetal or chromosomal anomalies. 3

### Weight Percentile Dropping Assessment

A decrease in fetal growth velocity, represented by a drop of 2 quartiles or 50 percentiles in EFW or AC, should alert to the possibility of restriction. 2

### Customized Curves for Fetal Growth

Universal curves assume that, under ideal conditions, all fetuses are expected to have the same growth potential, regardless of country of origin, race, and environmental factors. These are international curves developed from multicentric, multinational, longitudinal, and prospective studies, in which data from several countries were compiled. Examples of these curves are the Intergrowth 21st and the WHO curve. 8 9 It should be considered that genetic variations between racial groups interfere with the growth potential, and therefore, race-specific curves should be preferred. The National Institute of Child Health and Human Development (NICHD), for instance, has made separate curves for white, black, Hispanic, and Asian pregnant women. 1 The GROW software, which in addition to maternal weight, height, and parity, also considers the fetal sex for the construction of weight curves. 10 There is an even more individualized approach, which uses specific software to draw a growth velocity curve for each fetus, but it requires serial ultrasound assessments from the beginning of pregnancy.

## Classification

After the diagnosis of FGR, the differentiation between early and late FGR must be made, as there are two distinct types, with differences in their severity, association with pre-eclampsia, and natural history of fetal deterioration. 2 Basically, they have different clinical, ultrasonographic, and anatomopathological characteristics. 2 The limit of gestational age at diagnosis to define early and late would be around 32 to 34 weeks, but 32 weeks seems to be the optimal point. 2 Early FGR represents 20 to 30% of all growth restrictions, or 1% of pregnancies, and is associated with preeclampsia in 50% of cases, leading to a severe and early placental insufficiency, with a very small fetus and chronic fetal hypoxia, related to high perinatal morbidity and mortality. 4 Abnormal umbilical artery (UA) Doppler is common and also presents a sequence of typical Doppler velocimetry alterations, involving UA, middle cerebral artery (MCA) and ductus venous (DV). The greatest challenge in this setting is the management, aiming to achieve a better balance between the risk of leaving the fetus in the uterus and the complications related to prematurity. The main placental histopathological findings are poor placental implantation, abnormalities in spiral arteries, poor maternal vascular perfusion, and villous/multifocal infarcts. In these cases, there is chronic ischemia of the chorionic villi, impairing the secretion of placental growth factor (PlGF) and causing excessive release of soluble fms-like tyrosine kinase-1 (sFlt-1) by “syncytial nodes”, resulting in an increase in the sFit-1/ PlGF ratio, which would identify early FGR. Maternal cardiovascular status is represented by decreased cardiac output and increased peripheral vascular resistance. 2

Late FGR represents 70 to 80% of all growth restrictions, or 5 to 10% of pregnancies, and is associated with PE in 10% of cases. Placental insufficiency is mild, so the fetus is not necessarily very small, and normal umbilical artery Doppler is common. On the other hand, there is a high association with altered cerebroplacental ratio (CPR), and in 25% of cases, MCA vasodilation is found, suggesting chronic hypoxia. This condition is associated with possible intrapartum fetal distress and cerebral acidosis, with no sequence of deterioration in Doppler velocimetric parameters, which may suddenly evolve to damage or unexpected fetal death in term fetuses, evidencing a low tolerance of these fetuses to hypoxia. 4 The greater challenge in this setting is the diagnosis, not the management itself. Placental histopathological findings are less specific, mainly diffuse changes. Maternal cardiovascular status is represented by mild or absent findings. 2

## Management

In regard to the place of evaluation of this patient, the protocols have different opinions, but none makes hospitalization of the patient mandatory as soon as the diagnosis of FGR is made. FCOG advises that hospitalization is not routinely indicated for surveillance, but that the decision must depend on local protocols. ISUOG and ACOG do not cite criteria for hospitalization. SMFM advises to consider hospitalization when the fetus needs to be evaluated more than 3 times a week and sets as criteria absent end-diastolic flow in UA (AEDF-UA), reverse end-diastolic flow in UA (REDF-UA), or some more severe alteration. FIGO orients admission to the hospital once AEDF-UA is seen.

There are two pharmacologic approaches that are known to improve neonatal prognosis and are mentioned by all protocols: corticosteroid therapy to reduce neonatal respiratory complications and magnesium sulfate to reduce the incidence and severity of cerebral palsy. Regarding corticosteroid therapy, all five protocols guide its administration in pregnancies whose deliveries may occur before 34 weeks of gestational age (GA). SMFM and ACOG extend the administration of corticosteroid therapy up to 36 + 6 in women at risk of giving birth in the next 7 days who did not receive any course of corticosteroids, based on a randomized controlled study with 1427 neonates, conducted by Gyamfi-Bannerman et al., 11 in 2016, showing the reduction of neonatal respiratory complications after a course of corticosteroid therapy in late preterms. In regard to fetal neuroprotection with magnesium sulfate, there is still great divergence in the literature related to the upper limit of GA for its use. The FCOG protocol guides the administration in pregnancies whose deliveries are likely to happen before 32-33 weeks, ideally hours before delivery. ISUOG and FIGO advise that the use should be based on local protocols, given the heterogeneity of studies. SMFM and ACOG advise on births under 32 weeks.

### Surveillance Tools and Frequency

It is a consensus among all protocols that fetal weight assessment should not be performed less than 2 weeks apart. All protocols are unanimous regarding the multimodal assessment of the combination of Doppler velocimetry parameters (UA, MCA, and DV) with biophysical parameters such as cardiotocography (CTG), computerized cardiotocography (cCTG) and fetal biophysical profile (BP). Special attention is given to Doppler velocimetry and CTG, given the high availability of these methods. It is a consensus that the more severe the fetal alterations, the more frequently fetal vitality should be assessed.

CTG is a low complexity non-invasive biophysical method that is highly available around the world, including in low- and middle-income countries that shows a good correlation with fetal hypoxemia when it presents reduced variability. 12 Computerized cardiotocography (cCTG) has the same method of fetal vitality assessment, with the addition of providing objective and mathematical parameters to assess fetal vitality. 13 Therefore, it shows a significant increase in diagnostic and prognostic accuracy compared to conventional CTG in growth-restricted fetuses, especially below 32 weeks, but it is considerably less available around the world. 14 In 2014, Figueras and Gratacós 4 noted that there is no evidence to support the use of conventional CTG in fetuses with growth restriction because it has not shown benefits in reducing perinatal mortality due to the high rate of false positives, and cCTG would bring the benefit of being more accurate in detecting advanced fetal deterioration. In addition to Doppler velocimetry, CTG, and cCTG are the most used methods and are recommended by protocols for the surveillance of fetuses with FGR. The presence of some changes in CTG or cCTG is an absolute indication of termination of pregnancy, consistent in all protocols, such as the presence of persistent late decelerations in CTG, short-term variability (STV) <2.6ms up to 28 + 6 or STV <3ms up to 31 + 6.

The benefits of using Doppler velocimetry in high-risk pregnancies are very well documented, with a reduction in the risk of perinatal death and unnecessary obstetric interventions, especially with UA Doppler velocimetry in fetuses with early FGR and MCA in fetuses with late FGR. 15 16 UA Doppler provides important diagnostic and prognostic data in this context. Importantly, the absence of abnormalities on UA Doppler does not rule out the existence of placental dysfunction, especially in fetuses with late FGR. The worsening of increased resistance in UA, AEDF-UA, and REDF-UA can be slow and gradual in fetuses with FGR and demonstrate an important reduction in blood flow, is related to fetal deterioration, with perinatal morbidity and mortality rates being proportional with the degree of change on Doppler velocimetry. 16 17 The median time interval between deterioration from increased UA resistance to AEDF-UA is 2 weeks, between AEDF-UA and cardiovascular deterioration is 5 days (with a weighted odds ratio of 3.6 for fetal loss) and between REDF-UA and more severe fetal deterioration is 2 days (with a weighted odds ratio of 7.3 for fetal loss). 12

MCA Doppler velocimetry plays an important role, especially in fetuses with late FGR. Fetal hypoxemia resulting from cardiovascular alterations in fetuses with FGR leads to the activation of a vasodilation mechanism aimed at ensuring the supply of nutrients and oxygen to noble organs, such as the brain. 18 This cerebral vasodilation can be assessed using the pulsatility index (PI) of the MCA and the cerebroplacental ratio (CPR). The MCA Doppler has modest and inefficient prognostic values in regard to perinatal morbidity and mortality in fetuses with early FGR, but it has a better performance in the evaluation of fetuses with late FGR, especially CPR. 19 20 Based on a retrospective study conducted by Crimmins et al. 21 with 987 pregnancies complicated with FGR, the ISUOG and FIGO protocols reinforce UA and MCA Doppler guidance at least twice a week in fetuses with late FGR, since the median time between MCA vasodilation and stillbirth in this population was 5 days. On the other hand, it is worth mentioning that SMFM advocates against performing MCA and uterine arteries Doppler in the routine clinical management of FGR (grade 2B), based on meta-analyses and studies that showed no high degree of prediction of perinatal adverse outcomes in the assessment of these markers. 22 23 However, these last two studies did not subdivide the fetuses according to gestational ages and their results should be evaluated in light of this information.

DV Dopplervelocimetry provides information about the degree of cardiac involvement, once it reflects pressure-volume changes in the right atrium and it plays its most marked role in fetuses with early FGR, being a reliable guide for defining the ideal delivery time, in combination with cCTG in this setting. 24 25 Its routine performance is discouraged by the SMFM protocol after 32 weeks. 3 DV alterations have an important correlation with fetal hypoxemia and risk of intrauterine death. 26 The FCOG and FIGO protocols emphasize that DV Doppler should only be performed by trained operators and only in pregnancies with FGR whose interruption is likely to occur before 32 weeks. Chart 1 summarizes the recommendations on the frequency of examinations.

**Chart 1:** Frequency of fetal vitality assessment

	FIGO 1	ISUOG 2	SMFM 3	FCOG 5	ACOG 6
If no changes in Doppler	Doppler every 1-2 weeks	–	Doppler weekly	Doppler every 2-3 weeks	–
If early doppler changes (IP UA >p95 or IP MCA <p5)	Doppler 1-2 times a week and BP twice a week	–	Doppler weekly	Doppler weekly	–
If AEDF-UA	Doppler 3 times a week and BP daily	Doppler 2-3 times a week	Doppler 2-3 times a week and CTG daily	Doppler more than once a week and CTG daily	–
If REDF-UA	Doppler daily and BP twice a day	Doppler 2-3 times a week	CTG daily	Doppler more than once a week and CTG daily	–
If ductus venosus alteration (PI> p95, absent or reversed A wave)	Doppler daily and BP twice a day	–	–	–	–

AEDF-UA= absent end-diastolic flow in umbilical artery; BP= biophysical profile; CTG= cardiotocography; MCA= middle cerebral artery; p5= 5 ^th^ percentile; p95= 95 ^th^ percentile; PI= pulsatility index; REDF-UA= reverse end-diastolic flow in umbilical artery; UA= umbilical artery.

### Time and Mode of Delivery

The most important prognostic factor in fetuses with FGR is the gestational age at the delivery. 1 Thus, the risks and benefits of interrupting or maintaining the pregnancy must always be weighed, considering the probability of stillbirth or other adverse outcomes and the innate complications of prematurity. There is, in general, no unanimity or uniformity in the protocols regarding this point, mainly due to the absence of robust studies for these outcomes and the heterogeneity of the methodology of the existing studies. Chart 2 summarizes the main indications for termination of pregnancy and the gestational age recommended by each protocol. After reaching fetal viability, some situations are taken as absolute criteria for delivery, regardless of gestational age, such as spontaneous, late, and persistent decelerations in CTG, important and permanent decrease in variability in CTG, obstetric emergency, or absolute maternal indication. The most cited study by the protocols regarding the time of delivery was the multicenter study TRUFFLE (Trial of Randomized Umbilical and Fetal Flow in Europe), conducted between 2005 and 2010 with approximately 500 pregnant women with gestational age less than or equal to 32 weeks. 27 TRUFFLE evidenced the existence of better perinatal morbidity and mortality outcomes related to the creation of a standardized protocol for the management and diagnosis of FGR fetuses that associates with different modalities of fetal vitality assessment. In that study, in a 2-year follow-up after birth, termination of pregnancy based on late changes in the ductus venosus was the least associated with neurodevelopmental disorders, but with increased perinatal mortality rates compared to managed groups through data cCTG or early DV changes. The safety-net criteria for termination of pregnancy used by the TRUFFLE researchers are the same recommended by the major protocols, such as ISUOG and FIGO ( Chart 2 ).

**Chart 2:** Safety-net criteria for indication of delivery, regardless of the randomized group, in the TRUFFLE study

1-STV Criteria
a) STV <2.6ms between 26 + 0 and 28 + 6
b) STV <3.0ms between 29 + 0 and 31 + 6
c) Delivery regardless of STV if there are spontaneous, repeated and persistent decelerations in CTG
2- UA Doppler Criteria
a) AEDF-UA above 34 weeks
b) REDF-UA over 32 weeks
c) Following local protocols, delivery could be performed if AEDF-UA above 32 weeks or REDF-UA above 30 weeks

AEDF-UA= absent end-diastolic flow in umbilical artery; REDF-UA= reverse end-diastolic flow in umbilical artery; STV= short-term variability; UA= umbilcal artery.

Another frequently cited multicentric study is the GRIT (The Growth Restriction Intervention Trial), conducted by the GRIT Study Group in 13 European countries with women with fetuses with FGR below 36 weeks, published in 2003. 28 In this study, 547 pregnant women with signs of fetal vitality impairment in which the obstetricians were in doubt about the interruption of the pregnancy at that time were divided into two groups: immediate interruption or waiting until the obstetrician was no longer uncertain about the interruption. It was seen that there was no difference in perinatal survival rates or changes in neuropsychomotor development of these children at 2, 6, and 12 years of follow-up between the two groups.

Regarding the optimal time of interruption of preterm pregnancies and early-term fetuses, the ACOG protocol considers the guidelines drawn from some conclusions of a 2011 workshop produced by the Eunice Kennedy Shriver National Institute of Child Health and Human Development, by the Society for Maternal-Fetal Medicine and by the ACOG itself. 29 The only large study evaluating fetuses with FGR at term or late preterms was the Disproportionate Intrauterine Growth Intervention Trial At Term Study (DIGITAT), carried out with 650 pregnant women with growth-restricted fetuses divided into two groups: induction of labor or expectant management. 30 The fetuses in the first group had a mean gestational age at birth 10 days lower than those in the second group, but without statistically relevant differences regarding adverse perinatal outcomes (death before hospital discharge, 5-minute APGAR less than 7, fetal acidemia at birth or admission to the NICU) between the two groups. Based on this work, on the workshop mentioned above, and on the opinion of experts, the ACOG guides pregnancy interruption between 34 + 0 and 37 + 6 if FGR is associated with factors that increase the risk of adverse outcome (factors such as oligohydramnios, Doppler alteration, maternal risk factors or comorbidities) and up to 37 + 0 in fetuses with EFW below the 3rd percentile. 27 FIGO, in turn, based on the DIGITAT Study and in expert opinion, advises interruption between 36 and 38 weeks in fetuses with EFW below the 3rd percentile, between 34 and 37 weeks in fetuses with FGR and early Doppler changes or associated mild abnormalities (oligohydramnios, suboptimal growth, and pre-eclampsia).

The meta-analysis performed by Caradeux et al., 26 in 2018 supports the high perinatal mortality rates in fetuses with FGR below 34 weeks when there are specific changes in Doppler: overall risk for fetal death of 6.8% if AEF-UA, 19% in REDF-UA and 20% in absent or reverse a wave in DV. 26 This meta-analysis is also used as a reference for the interruption criteria in the FIGO and SMFM protocols. Chart 3 summarizes the recommendations for termination of pregnancy.

**Chart 3:** Recommendations on timing of termination of pregnancy in FGR and SGA fetuses

	FIGO 1	ISUOG 2	SMFM 3	FCOG 5	ACOG 6
EFW p3-p10, no changes on Doppler (SGA)	37 to 39 weeks	38 to 39 weeks	38 to 39 weeks	–	38 + 0 to 39 + 6 weeks
EFW <p3 (isolated FGR), no changes on Doppler	36 to 38 weeks	36 to 37 weeks	37 weeks	–	37 weeks
Early Doppler changes (PI UA >p95, PI MCA <p5, CPR <p5, mean PI UtA >p95)	34 to 37 weeks (including changes such as oligohydramnios and suboptimal growth)	36 + 0 to 37 + 6 weeks	37 weeks	–	–
AEDF-UA	32 weeks	34 weeks	33 to 34 weeks	–	–
REDF-UA	30 weeks	32 + 0 to 33 + 6 weeks	30 to 32 weeks	–	–
Changes in the ductus venosus (PI >p95, absent or reversed A wave)	26 weeks	26 weeks	26 weeks	–	–

AEDF-UA= absent end-diastolic flow in umibilical artery; EFW= estimated fetal weight; MCA= middle cerebral artery; p3= 3 ^rd^ percentile; p5= 5 ^th^ percentile; p95= 95 ^th^ percentile; PI= pulsatility index; REDF-UA= reverse end-diastolic flow in umbilical artery; UA= umbilcal artery; UtA= uterine arteries.

Regarding the mode of delivery, Cesarean section is consensually indicated in all protocols in cases of AEDF-UA of REDF-UA, if STV changes are present, if there is an maternal indication, or if the fetal status is not reassuring, with abnormalities in CTG or BP. In other cases, vaginal delivery can be induced, but with the indication of continuous intrapartum fetal monitoring. After delivery, FCOG and FIGO advise sending the placenta for an anatomopathological examination, which, despite not increasing the diagnostic accuracy, can provide information about the risk of FGR recurrence.

## Conclusion

The aim of this paper was to present, in a didactic way, the particularities of different protocols for monitoring fetuses with growth restriction, in order to help obstetricians to better manage the cases. As we could see, there is no flawless protocol and they all have complementary information in some way. Due to several variations in the management of FGR fetuses in protocols, there is no way to summarize all the orientations of all the guidelines on the management of fetal FGR. Thus, it is important that health professionals carefully examine the protocols, analyze and interpret the studies so that they could use them as a basis for formulating their own protocols, considering local realities.
